# First person – Jessika Wise

**DOI:** 10.1242/bio.062539

**Published:** 2026-03-03

## Abstract

First Person is a series of interviews with the first authors of a selection of papers published in Biology Open, helping researchers promote themselves alongside their papers. Jessika Wise is first author on ‘ Gelatin-methacryloyl hydrogel stiffness influences epithelial-mesenchymal transition in MCF7 but not MDA-MB-231 breast cancer cells in 3D culture’, published in BiO. Jessika conducted the research described in this article while a PhD candidate under co-supervision by Dr Elisabeth Phillips, Professor Khoon Lim, Professor Tim Woodfield and Associate Professor Margaret Currie at University of Otago Christchurch, Christchurch, New Zealand. She is now a Laboratory Manager/Research Fellow in the labs of Associate Professor Anna Pilbrow and Professor Chris Pemberton at the University of Otago Christchurch, where her research focuses on cardiovascular biomarker discovery and development and human cellular models of ischaemia-reperfusion injury in acute coronary syndromes.


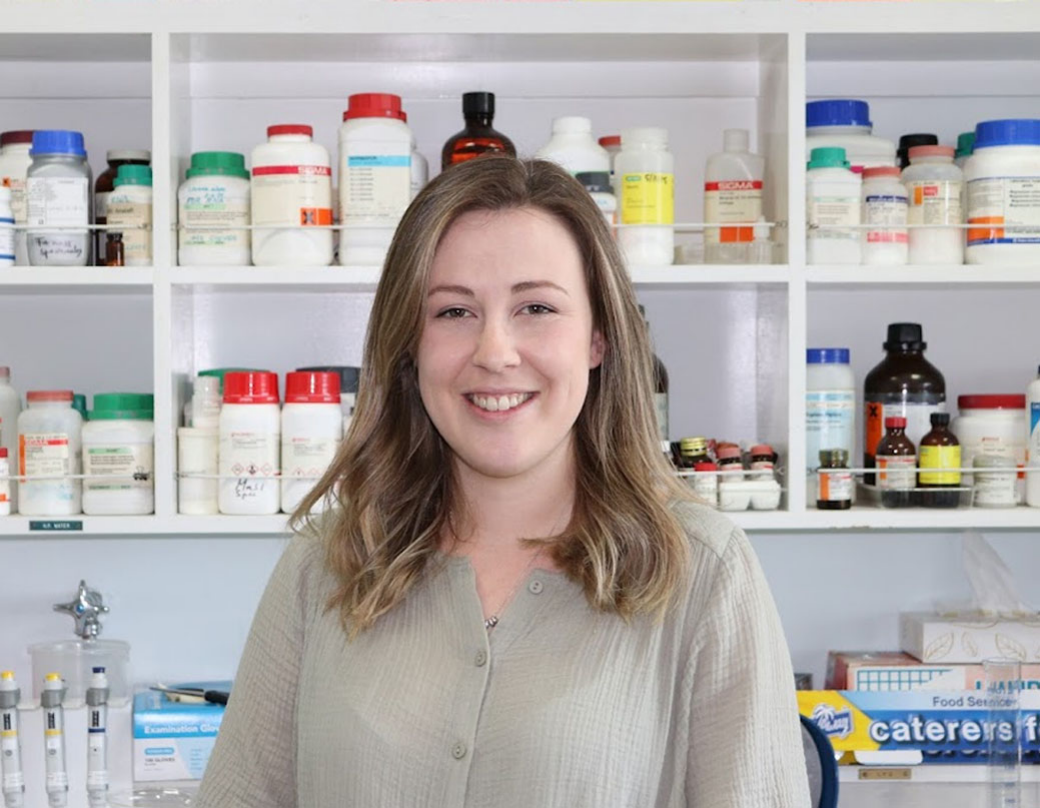


**Jessika Wise**

**Describe your scientific journey and your current research focus**

My first hands-on research experience was during my BSc (Honours) at the University of Otago with Dr Elspeth Gold, where I used transwell co-culture models to explore how prostate cancer cells interact with healthy stromal cells and respond to growth inhibitors. This work sparked my fascination with how cells communicate in complex environments, which I carried into my PhD at the University of Otago Christchurch. My doctoral research, a collaboration between the Mackenzie Cancer Research Group and the Christchurch Regenerative Medicine and Tissue Engineering (CReaTE) group, involved developing a 3D co-culture model combining breast cancer cells with patient-derived mature adipocytes to study tumour-microenvironment interactions.

After completing my PhD, I had the exciting opportunity to work in the biotechnology sector with Upstream Medical Technologies, focusing on cardiovascular biomarker discovery to support clinical diagnostics in Aotearoa New Zealand. That experience opened doors to my current roles as Laboratory Manager and Research Fellow at the Christchurch Heart Institute, University of Otago Christchurch, where I now use human cellular models to study biomarkers, cardiac ischaemia/reperfusion injury, and the genetic and epigenetic drivers of cardiovascular disease.

**Who or what inspired you to become a scientist?**

Throughout my education, I have been fortunate to have teachers who made science not only fascinating but clearly relevant to everyday life. In high school, I was taught by two science teachers with PhDs who brought research thinking into the classroom. A particularly important influence was the late Dr Elspeth Gold. Her infectious enthusiasm for every new result and her unwavering support for students made science feel both exciting and attainable. She demonstrated the thrill of discovery and showed me that being a scientist is as much about curiosity and perseverance as it is about technical skill. Today, I am inspired by my colleagues at the Christchurch Heart Institute, who I was fortunate to work alongside during my time with Upstream Medical Technologies and now collaborate with directly within the Institute itself. Their dedication, generosity with their knowledge and commitment to improving cardiovascular health reinforce that meaningful science is always connected to people. Working alongside them has shaped the kind of scientist I want to be.

… the ‘stiffness’ of a tumour's environment can influence how cancer progresses

**How would you explain the main finding of your paper?**

We studied how the environment around breast cancer cells affects their behaviour. We found that stiffer surroundings can make some cancer cells change in ways that make them more likely to spread, while softer surroundings don't. This shows that the ‘stiffness’ of a tumour's environment can influence how cancer progresses, and our 3D models give a realistic way to study this.

**Figure:**
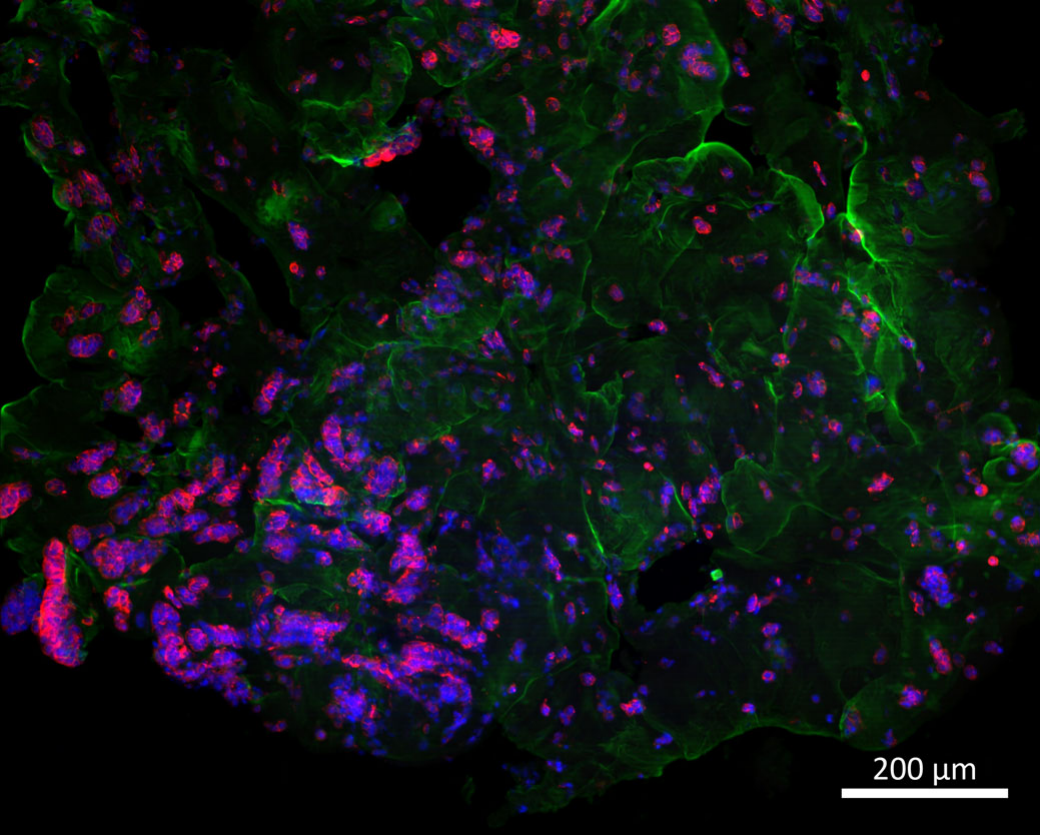
**Multicellular clustering of MCF7 breast cancer cells within a soft gelatin-methacryloyl matrix.** Collagen I highlights the hydrogel structure (green), F-actin visualises cell morphology (red) and DAPI labels nuclei (blue).

**What are the potential implications of this finding for your field of research?**

These findings help us understand how the physical environment around a tumour can influence cancer progression. By showing that stiffness can drive changes in cancer cells, this work highlights a factor that could be targeted in future therapies or considered when developing more realistic lab models. More broadly, it suggests that studying the tumour environment, not just the cancer cells themselves, is important for understanding disease behaviour and testing potential treatments.

**What do you enjoy most about being an early-career researcher?**

One of the things I enjoy the most as an early-career researcher is having the freedom to explore new ideas while learning from experienced colleagues. I appreciate the balance of independence and mentorship, and the collaborative environment that allows for problem solving alongside others.

**What piece of advice would you give to the next generation of researchers?**

I would tell them to remember the bigger picture of their work. Understanding how your research could ultimately improve healthcare or people's lives is a rare opportunity, and keeping that perspective can guide decisions, inspire creativity and make the most challenging parts of research feel worthwhile.
